# Phosphatidylserine-selective targeting and anticancer effects of SapC-DOPS nanovesicles on brain tumors

**DOI:** 10.18632/oncotarget.2214

**Published:** 2014-07-14

**Authors:** Víctor M. Blanco, Zhengtao Chu, Subrahmanya D. Vallabhapurapu, Mahaboob K. Sulaiman, Ady Kendler, Olivier Rixe, Ronald E. Warnick, Robert S. Franco, Xiaoyang Qi

**Affiliations:** ^1^ Division of Hematology and Oncology, Department of Internal Medicine, University of Cincinnati College of Medicine, Cincinnati, Ohio; ^2^ Division of Human Genetics, Department of Pediatrics, Cincinnati Children's Hospital Medical Center, Cincinnati, Ohio; ^3^ Department of Pathology and Laboratory Medicine, University of Cincinnati College of Medicine, Cincinnati, Ohio; ^4^ Division of Hematology/Oncology, Georgia Regents University, GRU Cancer Center, Augusta, Georgia; ^5^ Department of Neurosurgery, University of Cincinnati Brain Tumor Center, and Mayfield Clinic, Cincinnati, Ohio

**Keywords:** Glioblastoma, brain metastasis, SapC-DOPS, imaging, cancer therapy

## Abstract

Brain tumors, either primary (e.g., glioblastoma multiforme) or secondary (metastatic), remain among the most intractable and fatal of all cancers. We have shown that nanovesicles consisting of Saposin C (SapC) and dioleylphosphatidylserine (DOPS) are able to effectively target and kill cancer cells both *in vitro* and *in vivo*. These actions are a consequence of the affinity of SapC-DOPS for phosphatidylserine, an acidic phospholipid abundantly present in the outer membrane of a variety of tumor cells and tumor-associated vasculature. In this study, we first characterize SapC-DOPS bioavailability and antitumor effects on human glioblastoma xenografts, and confirm SapC-DOPS specificity towards phosphatidylserine by showing that glioblastoma targeting is abrogated after *in vivo* exposure to lactadherin, which binds phosphatidylserine with high affinity. Second, we demonstrate that SapC-DOPS selectively targets brain metastases-forming cancer cells both *in vitro*, in co-cultures with human astrocytes, and *in vivo*, in mouse models of brain metastases derived from human breast or lung cancer cells. Third, we demonstrate that SapC-DOPS nanovesicles have cytotoxic activity against metastatic breast cancer cells *in vitro*, and prolong the survival of mice harboring brain metastases. Taken together, these results support the potential of SapC-DOPS for the diagnosis and therapy of primary and metastatic brain tumors.

## INTRODUCTION

Standard cancer therapies that often show good results in systemic cancers usually fail to deter the progression of brain tumors [1, 2]. As a consequence, compared with most extracranial tumors, the prognosis for both glioblastoma multiforme (GBM), the most aggressive and prevalent malignant brain tumor, and for secondary (metastatic) brain tumors is very poor, with a median survival of one year or less [3, 4]. Primary brain tumors are the second leading cause of cancer-related death in males up to the age of 40, and approximately 22,000 new cases of malignant brain tumors were detected in the US per year between 2006 and 2010 [5]. Unfortunately, a large number of clinical trials have produced only modest benefits regarding GBM recurrence prevention and patient survival [6]. Brain metastases arise mainly from primary lung, skin, and breast cancers, affect 10-30% of adult cancer patients and are much more prevalent than primary brain tumors [7]. Although new targeted therapies are showing promising results [8], the difficulty in diagnosing and effectively targeting small micrometastases makes the treatment of brain metastases one of the most pressing challenges in clinical oncology.

We developed a nanovesicle composed of two naturally-occurring molecules, saposin C (SapC)–dioleylphosphatidylserine (DOPS) that show diagnostic and antitumor capabilities both *in vitro* and *in vivo* [9-11]. Saposin C (SapC) is a small (80 amino acids), stable, acidic lysosomal protein with fusogenic affinity for phosphatidylserine-enriched membranes that functions as a critical co-activator of lysosomal enzymes such as sphingomyelinase and acid beta-glucosidase [12, 13]. Increased exposure of phosphatidylserine characterizes the membrane surfaces of tumor cells and tumor-associated vasculatures [14, 15], and represents an attractive target for anticancer therapies [16, 17]. Previous work from our lab has shown that fusion of SapC-DOPS to cancer cells led to apoptotic death due to ceramide accumulation and caspase activation [9, 11, 18]. Recently, we showed that SapC-DOPS has anti-angiogenic and pro-survival effects in GBM mouse models [11] and can also target spontaneous metastases of orthotopically-implanted pancreatic cancer cells [18].

In this study, we evaluate SapC-DOPS bioavailability in orthotopic GBM xenografts, assess its effectiveness against the growth of subcutaneous GBM xenografts, and address its ability to selectively target brain metastases of human breast and lung cancer cells in mice. To determine the kinetics of SapC-DOPS uptake by GBM cells *in vivo*, we evaluated its accumulation for up to 24 hours post-systemic injection. Having recently proved SapC-DOPS efficacy against orthotopic U87ΔEGFR GBM tumor xenografts with constitutive EGFR activation [11], in this report we assessed SapC-DOPS' antitumor actions on non-genetically engineered U87-MG GBM cell xenografts. Then, using co-cultures of human astrocytes and human breast cancer cells with brain metastatic potential, we assessed the binding selectivity of SapC-DOPS, and characterized its tumor cytotoxicity *in vitro*. Finally, we demonstrate the ability of SapC-DOPS nanovesicles to selectively target and label small brain metastases derived from human breast and lung primary cancer cells in mice *in vivo*, and assess the therapeutic benefits of SapC-DOPS in mice with brain metastases of breast cancer cells. Taken together, our findings highlight the potential of SapC-DOPS nanovesicles in the diagnosis and therapy of both primary and metastatic CNS cancers.

## RESULTS

### Kinetics of SapC-DOPS targeting and uptake in glioblastoma *in vivo*

**Figure 1:**
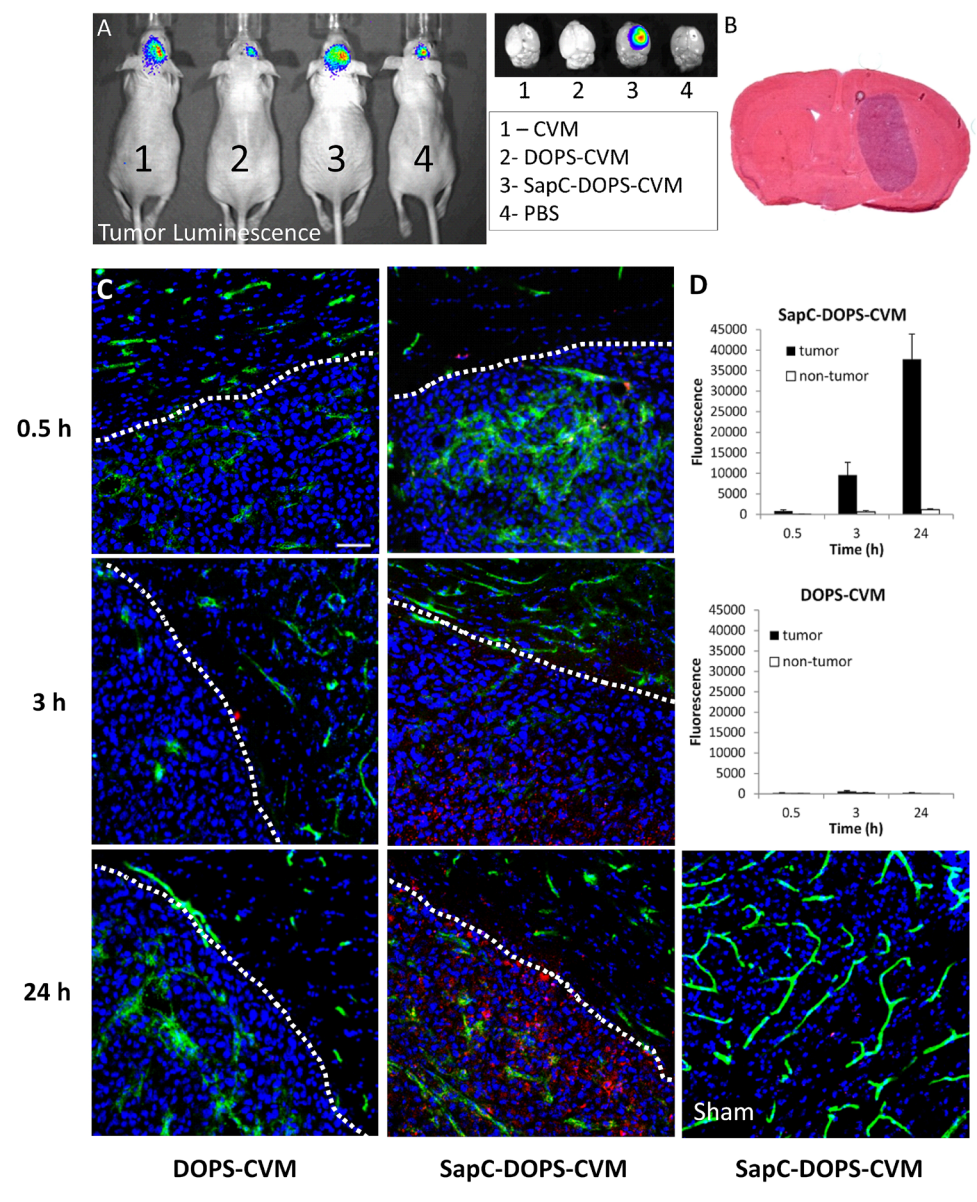
Time-dependent accumulation of SapC-DOPS in glioblastomas (A) *In vivo* bioluminescence imaging of orthotopic U87ΔEGFR-Luc xenografts. Shown on right side are the dissected brains subjected to fluorescence imaging 24 h after injection with CVM, DOPS-CVM, SapC-DOPS-CVM, or PBS. (B) H&E staining of a mouse brain section harboring a U87ΔEGFR-Luc tumor. (C) Confocal fluorescence microscopy of U87ΔEGFR-Luc tumors and adjacent normal brain parenchyma. DOPS-CVM or SapC-DOPS-CVM (red signal) was injected i.v and the mice sacrificed after 0.5, 3 or 24 hs. (D) Quantification of DOPS-CVM and SapC-DOPS-CVM signal intensity in tumor regions and adjacent brain parenchyma (non-tumor) from microphotographs such as those shown in C; n = 3 mice per treatment. Scale bar = 50 μm.

We recently showed that SapC-DOPS nanovesicles inhibit tumor angiogenesis and improve the outcome of GBM-bearing mice [11]. In order to maximize the diagnostic and therapeutic potential of SapC-DOPS, we characterized their targeting efficacy and bioavailability in mice intracranially implanted with human U87ΔEGFR-Luc GBM cells. Orthotopic implantation of these cells in athymic mice produced solid tumors (Fig. 1A and 1B) that led to significant morbidity and mortality after 12-16 days. After a single, systemic intravenous (tail vein) injection, fluorescently labeled SapC-DOPS (SapC-DOPS-CVM) accumulated selectively in these tumors (Fig. 1A), while no signal was observed in mice injected with PBS, DOPS-CVM, or CVM alone.

The time course of SapC-DOPS-CVM and DOPS-CVM accumulation in intracranial GBM xenografts was determined 30 min, 3 h, and 24h after intravenous injection. At these time points, mice were sacrificed and CVM fluorescence (red) was quantitated from confocal images of brain tissue sections (Fig. 1C). A progressive accumulation of SapC-DOPS-CVM was observed in GBM tumors; there was minimal intra-tumor accumulation of DOPS-CVM, while no signal was observed for either DOPS-CVM or SapC-DOPS-CVM in the brains of sham (PBS-injected) animals (Fig. 1D).

### SapC-DOPS crosses the blood-brain-tumor-barrier

**Figure 2:**
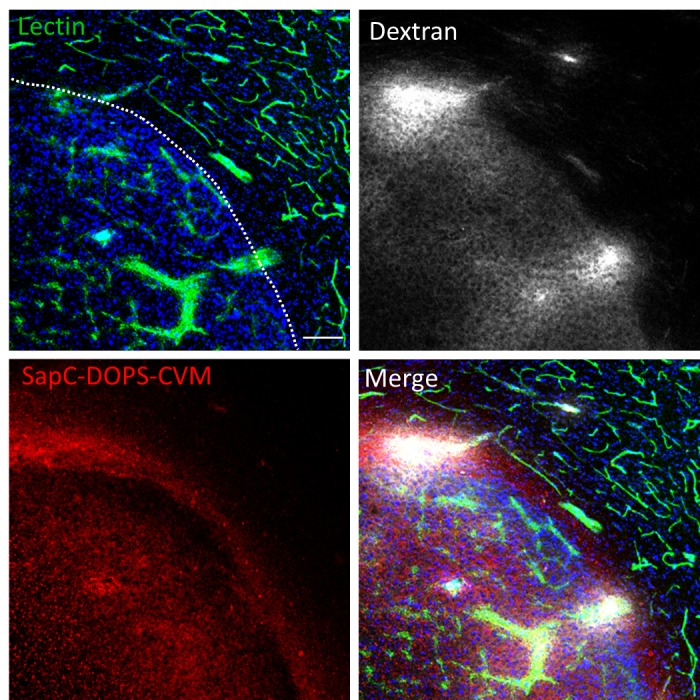
Glioblastoma vascularization and permeability Mouse brain section imaged with confocal microscopy showing a U87ΔEGFR-Luc tumor and adjacent normal brain parenchyma. Vascular structures (lectin-FITC), vascular permeability (dextran-TRITC) and intra-tumor SapC-DOPS-CVM accumulation were simultaneously assessed. Lectin-FITC, Dextran-TRITC and SapC-DOPS-CVM were injected i.v. 2 min, 30 min and 48 hs before sacrifice, respectively. Scale bar = 100 μm.

Aberrant angiogenesis and enhanced vascular permeability characterize many solid tumors in the brain as well as in peripheral organs, and may contribute to the tumor-selective targeting capacity of SapC-DOPS in our GBM model. We used fluorescently labeled lectin, dextran and SapC-DOPS to simultaneously assess vascular structure, vascular permeability and SapC-DOPS uptake in orthotopic U87ΔEGFR-Luc GBM in mice. As shown in Fig. 2, *in vivo* lectin-FITC staining revealed an enlarged, irregular tumor vasculature, while extravasation of dextran-TRITC (70 kDa) evidenced its marked permeability. By 48 hs after intravenous injection, SapC-DOPS-CVM extensively and specifically accumulated within the tumor. Incomplete colocalization of SapC-DOPS-CVM and lectin-FITC indicated that SapC-DOPS accumulated in the extravascular tumor space, thus confirming that SapC-DOPS nanovesicles are able to cross the blood-brain-tumor-barrier, but not the intact brain endothelium.

### SapC-DOPS targeting is mediated by exposed phosphatidylserine in tumor cells

**Figure 3:**
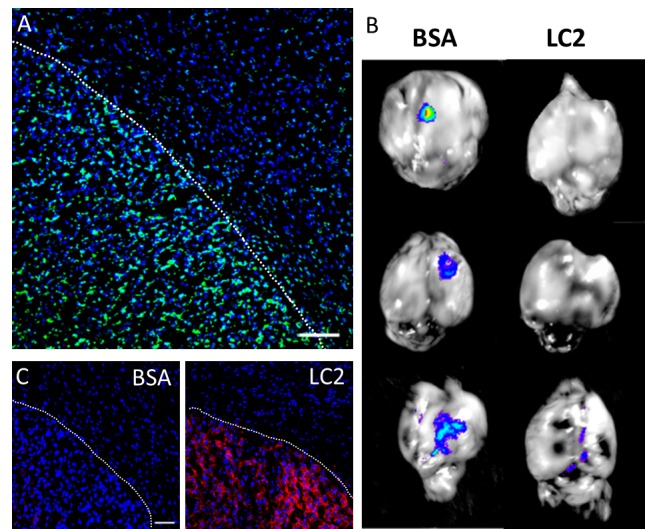
Phosphatidylserine expression in glioblastoma cells mediates intratumoral SapC-DOPS accumulation (A) Prominent phosphatidylserine (PS) immunoreactivity in U87ΔEGFR-Luc GBM in a mouse brain section. (B) SapC-DOPS-CVM imaging of mouse brains harboring U87ΔEGFR-Luc tumors. SapC-DOPS-CVM was injected 30 min after i.v. administration of BSA (control) or lactadherin (LC2). Pretreatment with LC2, which binds phosphatidylserine with high affinity, abolished or attenuated tumor targeting by SapC-DOPS. (C) Anti-LC2 immunofluorescence in brains of mice injected with BSA or LC2 demonstrates specific tumor localization of LC2. Scale bars = 100 μm.

Next, we assessed the importance of tumor phosphatidylserine expression on the selective uptake of SapC-DOPS by GBM *in vivo*. First, we performed immunostaining of intracranial U87ΔEGFR-Luc GBMs with an anti- phosphatidylserine antibody, which revealed strong tumor immunoreactivity, as compared with that of cells in the adjacent, normal brain parenchyma (Fig. 3A). Then, we tested the ability of lactadherin, a protein with high affinity for phosphatidylserine, to block SapC-DOPS targeting *in vivo*. To this end, GBM-bearing mice were injected intravenously with lactadherin or BSA (10 μg) 30 min before administration of SapC-DOPS-CVM. After 3 hours, *ex-vivo* brain imaging showed that SapC-DOPS-CVM targeting was nearly abolished in the three mice pretreated with lactadherin (Fig. 3B). Immunofluorescence staining confirmed that lactadherin localized to tumor cells and not to the surrounding brain stroma (Fig. 3C). These results suggest that SapC-DOPS binding to U87ΔEGFR-Luc GBM cells *in vivo* is mainly determined by phosphatidylserine exposure in these cells.

### Antitumor effects of SapC-DOPS on glioblastoma xenografts

**Figure 4:**
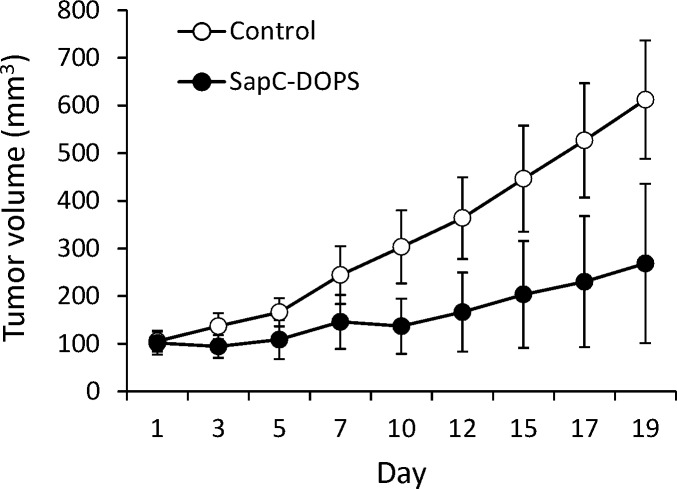
SapC-DOPS inhibits glioblastoma growth *in vivo* Nude mice (6 per group) were implanted with subcutaneous U87-MG xenografts and tumor size quantified by caliper measurement during systemic treatment with saline (Control) or SapC-DOPS.

In a previous report we showed the ability of SapC-DOPS to significantly prolong the survival of mice harboring orthotopic U87ΔEGFR-Luc GBM tumors that expressed the EGFRvIII mutation [11]. Here we sought to determine whether SapC-DOPS would also be effective against GBM tumors expressing the wild-type EGFR. To this end, nude mice were implanted subcutaneously with U87-MG cells, and when tumors reached ~100 mm^3^ mice were injected with a saline solution (control) or with SapC-DOPS (4 mg SapC/kg) daily for 7 days and every 2 days thereafter for 10 days. SapC-DOPS administration inhibited tumor growth by 56% (Fig. 4, n = 6, P < 0.05)

### SapC-DOPS selectively targets human cancer cells with metastatic potential

**Figure 5:**
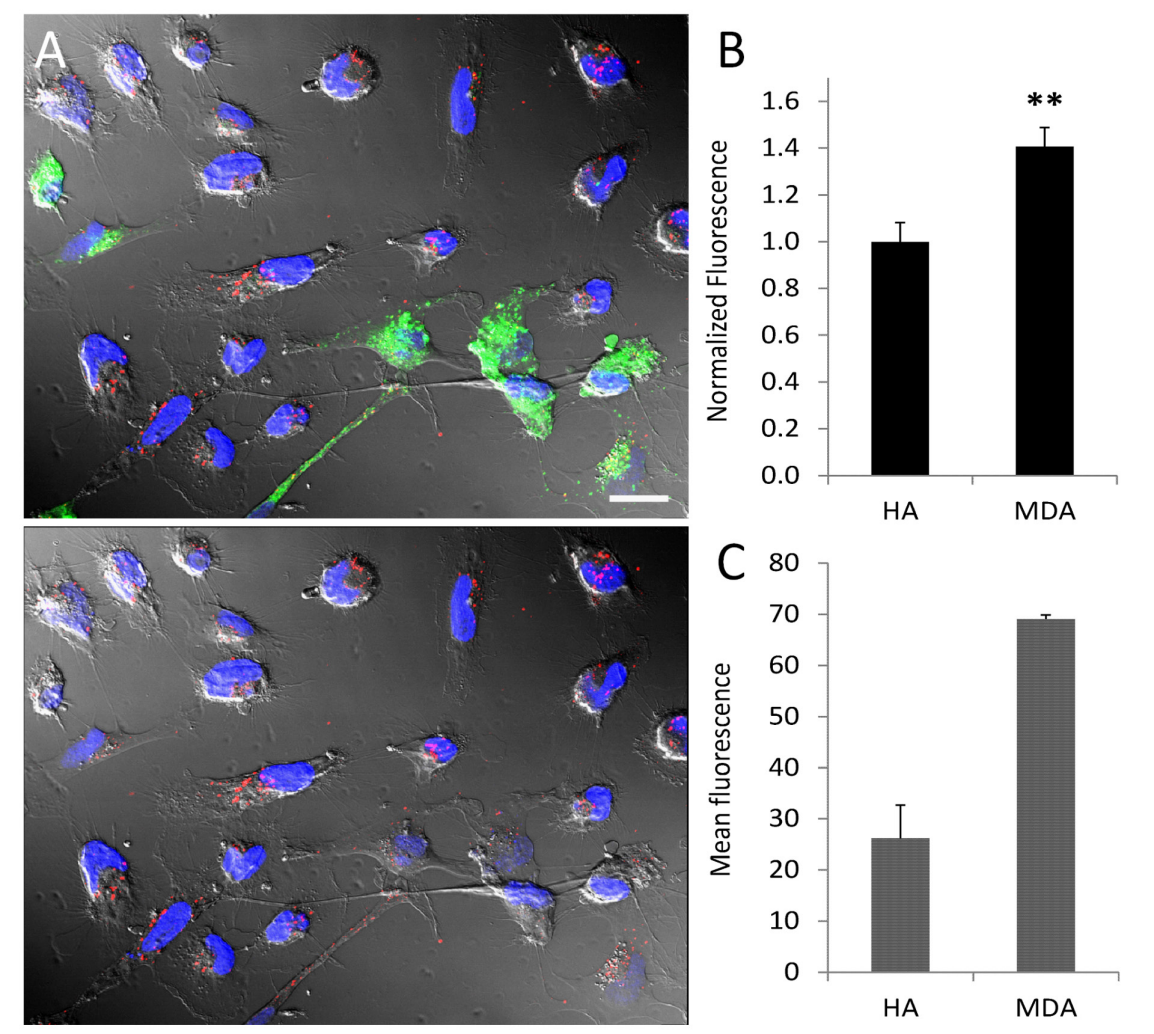
Tumor selective targeting of SapC-DOPS-CVM in co-cultured astrocytes and tumor cells (A) Normal human astrocytes and MDA-MB-231-luc-D3H2LN co-culture. Astrocytes (green) were labeled with PKH67. Cultures were exposed to SapC-DOPS-CVM (red) for 30 min. In the image at the bottom PKH67 fluorescence was omitted. (B) Quantification of SapC-DOPS-CVM uptake in co-cultured cells (pooled data from 10 microphotographs per culture; n=3 cultures). (C) Phosphatidylserine expression in cultured human astrocytes and MDA-MB-231-luc-D3H2LN cells, as assessed by flow cytometry using annexin V-FITC; n=3 cultures. **p<0.001. Scale bar = 20 μm.

After characterizing the targeting efficacy and bioavailability of SapC-DOPS in a primary brain cancer (GBM) model, we evaluated its ability both *in vitro* and *in vivo* to target cancer cells with brain metastasis potential. To this end, we first established an *in vitro* co-culture system comprising normal human astrocytes (HA) and metastatic breast cancer (MDA-MB-231-luc-D3H2LN) cells and compared SapC-DOPS uptake in both cell types after short (30 min) incubation. Fig. 5 illustrates and summarizes SapC-DOPS-CVM uptake (A; red fluorescence) and quantification data (B) for both cell types. Binding and uptake of SapC-DOPS-CVM was significantly higher in cancer cells. In line with the results presented above, this selectivity is likely to be a consequence of the elevated phosphatidylserine exposure levels exhibited by MDA-MB-231-luc-D3H2LN cells, as compared with those of normal astrocytes (Fig. 5C). Next, we evaluated the cytotoxicity of SapC-DOPS toward MDA-MB-231-luc-D3H2LN cells using the MTT cell viability assay. After 72 hs treatment, the assay revealed that SapC-DOPS killed tumor cells with an IC50 of 25.2 ± 1.5 μM (n = 3).

### SapC-DOPS targets brain micrometastases of human breast and lung cancer cells

**Figure 6:**
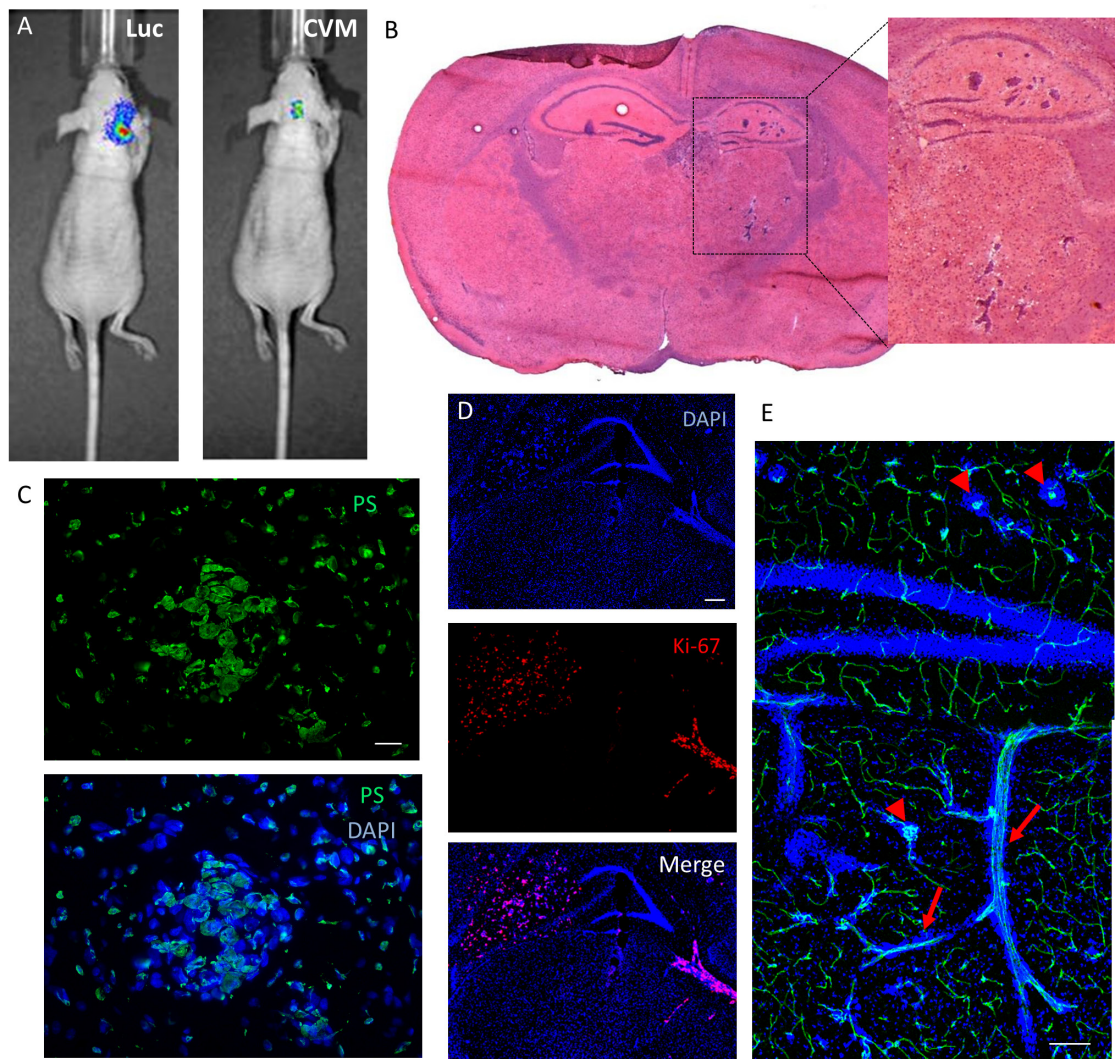
Brain metastasis mouse model: SapC-DOPS-targeting and anatomical and molecular features (A) Tumor cell luminescence (Luc) and SapC-DOPS-CVM fluorescence colocalize *in vivo*. SapC-DOPS-CVM was injected via the tail vein and the mouse imaged 24 hs later. B) H&E staining of a metastatic mouse brain. (C) Phosphatidylserine (PS) expression in a micrometastasis, as assessed by immunofluorescence in a mouse brain section. (D) Immunofluorescence against the mitosis marker Ki-67 (human) reveals active proliferation of MDA-MB-231-luc-D3H2LN cells in a mouse brain section. (E) Perivascular growth of breast cancer-derived MDA-MB-231-luc-D3H2LN cells in a mouse brain. Both capillary remodeling (arrowheads) and vessel co-option (arrows) characterize tumor cell growth. Scale bars = 25 μm (C); 200 μm (D, E).

The ability of SapC-DOPS to target brain metastases was evaluated in two metastatic brain tumor models, generated by injecting human breast cancer cells (MDA-MB-231-luc-D3H2LN) or human lung cancer cells (NCI-H460) into the internal carotid artery of nude mice. Fig. 6A shows an example of the luciferase bioluminescent signal (Luc) detected 51 days after injection of MDA-MB-231-luc-D3H2LN cancer cells in a live mouse, indicating the presence of tumor cells in the brain. Overlapping SapC-DOPS-CVM fluorescence, registered after a single intravenous injection, was indicative of SapC-DOPS's specific targeting of tumor cells (Fig. 6A, right side). Brain histology in these mice revealed multifocal micro- and macrometastases of epithelial morphology located predominantly in the hippocampal region (Fig. 6B); in some cases, metastatic cells were also found in the cortex, basal ganglia, and cerebellum. These cells strongly expressed phosphatidylserine (Fig. 6C) and evidenced extensive mitosis, as shown by immunostaining against the proliferation marker Ki-67 (Fig. 6D). Vascular labeling with lectin-FITC showed that MDA-MB-231-luc-D3H2LN tumor cells grow both as compact clusters, surrounding capillary structures, and also along preexisting vessels (Fig. 6E).

**Figure 7:**
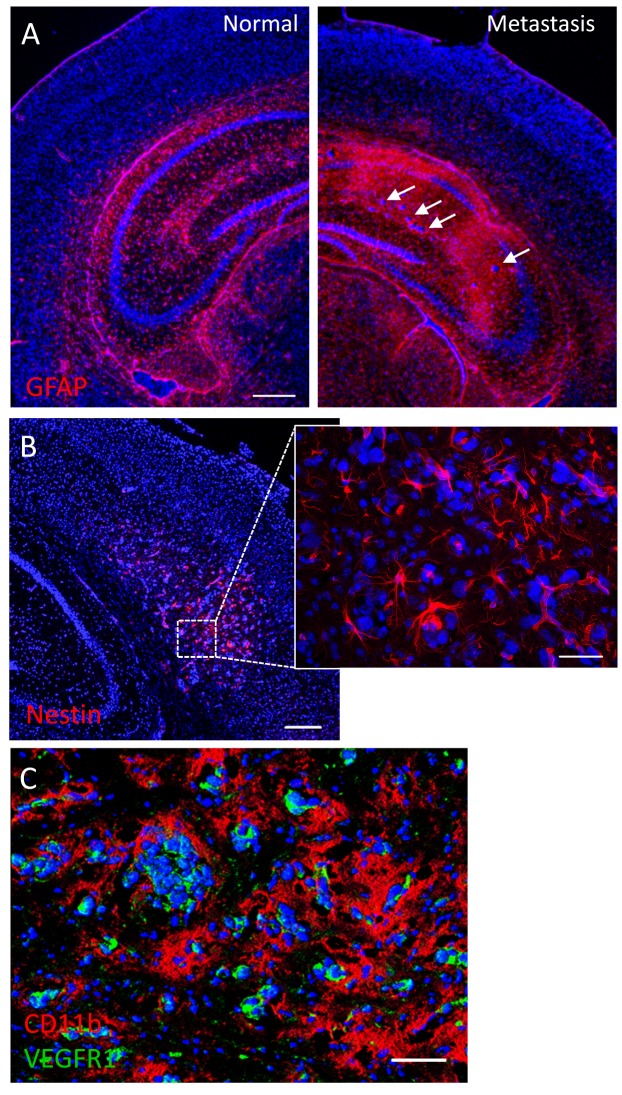
Tumor-stromal markers of brain metastasis (A) Immunofluorescence against the astrocytic marker GFAP shows extensive glial reactivity in a hippocampus harboring brain micrometastases (arrows). The contralateral hippocampus shows normal GFAP staining pattern. (B) Mouse nestin immunolabeling reveals numerous nestin-positive host cells with glial or endothelial morphologies in a brain region colonized by metastatic cancer cells. (C) Double immunofluorescence staining against CD11b and VEGFR1 in a metastatic mouse brain. Note the extensive coverage of cancer cells by CD11b-positive microglia/macrophages, while VEGFR1 expression seems to be restricted to metastatic cells. Scale bars = 200 μm (A, B); 20 μm (B, inset); 50 μm (C).

To better characterize this brain metastasis model, we performed immunohistochemistry to define potential tumor-stromal interactions. Brain regions affected with metastases showed marked astrogliosis as evidenced by GFAP immunostaining (Fig. 7A). Nestin-expressing host cells with stellate morphology were also prominently observed in close proximity to tumor cells (Fig. 7B); nestin expression sometimes overlapped with vascular (lectin) stain (not shown), suggesting a possible role of nestin-positive host cells in tumor vasculogenesis. Tumor cells were also tightly surrounded by CD11b-expressing myeloid cells and stained positively for the VEGF receptor VEGFR1 (Fig. 7C).

**Figure 8:**
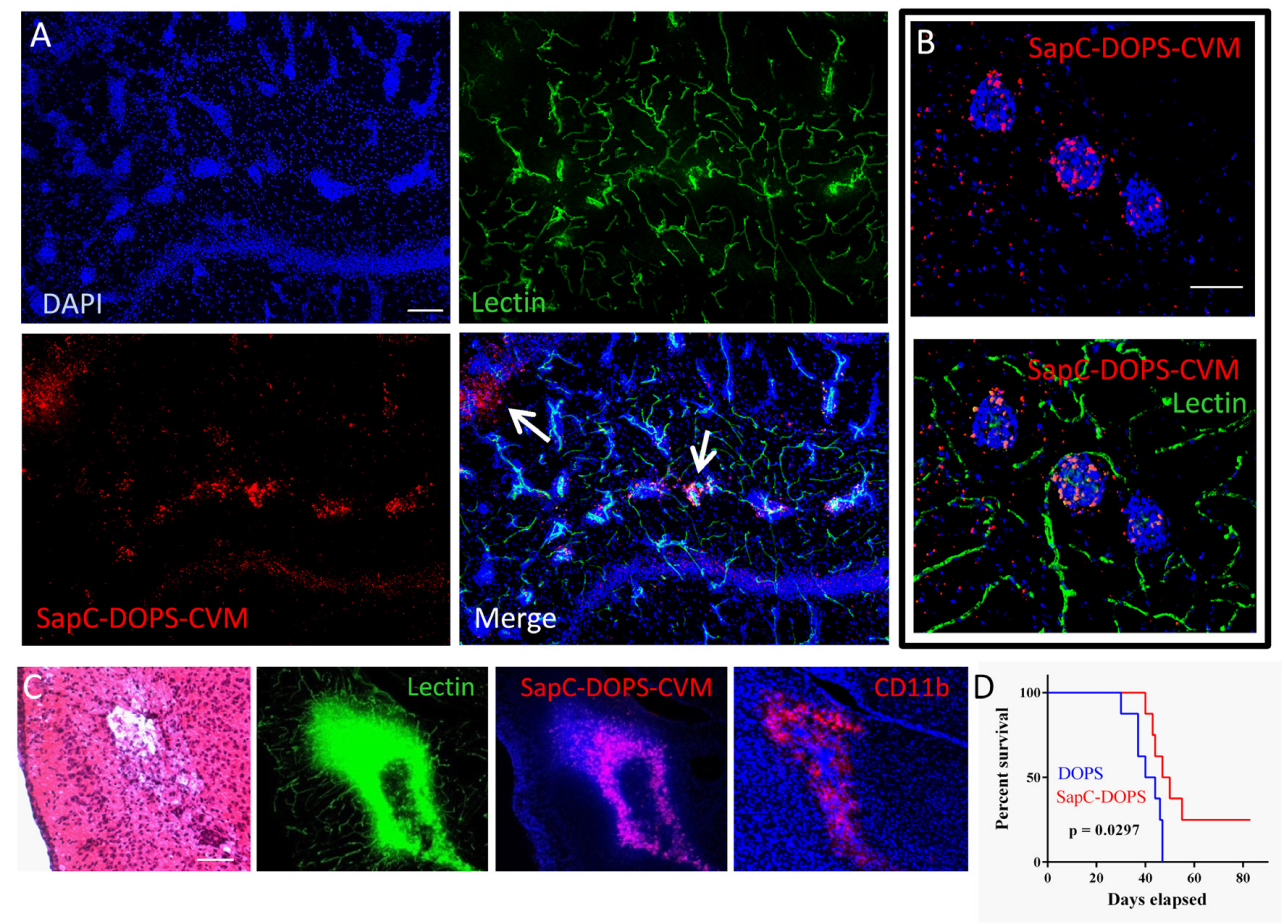
SapC-DOPS target brain metastases of breast cancer cells (A) Confocal picture showing SapC-DOPS-CVM targeting of brain micrometastases. SapC-DOPS-CVM was injected into the tail vein 24 hs before sacrifice; lectin-FITC was used to label the vasculature. Arrows point to SapC-DOPS-CVM that extravasated from the tumor capillaries into the parenchyma. Pictures are representative of results observed in 6 mice. (B) An example of SapC-DOPS-CVM targeting of the capillary network feeding brain micrometastases. (C) Consecutive sections of metastatic mouse brain tissue reveal regions of significant vascular damage associated with myeloid cell aggregates (CD11b) that are targeted by systemically injected SapC-DOPS-CVM. (D) Kaplan-Meier survival curves of mice treated with DOPS or SapC-DOPS (8 per group) after intracarotid injection with MDA-MB-231-luc-D3H2LN cells to induce brain metastases. Scale bars = 200 μm (A, C); 50 μm (B).

By 24 h after SapC-DOPS-CVM injection, fluorescence microscopy of brain sections showed that the nanovesicles targeted MDA-MB-231-luc-D3H2LN micrometastases, especially those exhibiting rich capillary remodeling (Fig. 8A and B). Extravascular SapC-DOPS-CVM signal was also apparent in some metastatic foci (arrows in Fig. 8A). Interestingly, we noticed that SapC-DOPS-CVM signal was conspicuously present nearby metastatic cell clusters in regions of vascular disruption, characterized by cell infiltrates that stained positively for the myeloid marker CD11b (Fig. 8C). As with the GBM model, SapC-DOPS-CVM fluorescence was absent in the intact brain parenchyma.

We next assessed the therapeutic effects of SapC-DOPS in mice harboring brain metastases. 5 days after intracarotid injection with MDA-MB-231-luc-D3H2LN cells, animals (8 per group) received either DOPS or SapC-DOPS injections (daily for 7 days, then every 2 days for more 10 more days). Mice treated with SapC-DOPS showed significantly extended survival compared with those treated with DOPS (Figure 8D; n = 8; p < 0.05).

**Figure 9:**
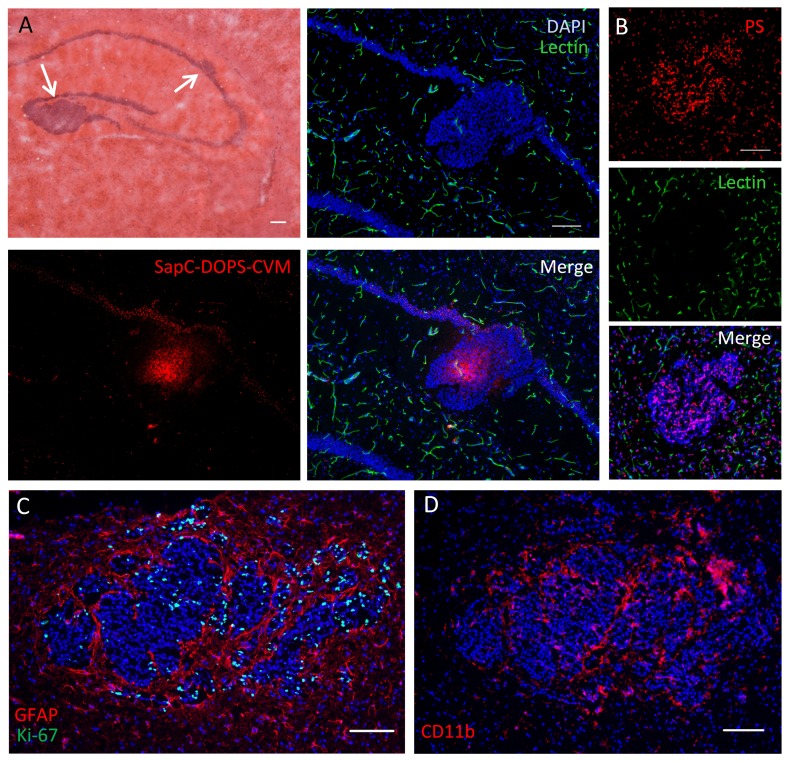
SapC-DOPS target brain metastases of lung cancer cells (A) NCI-H460 lung carcinoma cell clusters in the mouse hippocampus visualized with hematoxylin and eosin staining (top left). Contiguous fluorescence images illustrate the selective targeting of tumor cells by SapC-DOPS-CVM, 24 h after systemic administration; green structures are blood vessels stained with lectin-FITC. Pictures are representative of results observed in 4 mice. (B) Phosphatidylserine (PS) immunostaining of metastatic lung cancer cells. Lectin-FITC staining is shown here to demonstrate the scarcity of vascular elements inside the tumor mass. (C) Dual immunostaining for GFAP and Ki-67 in a tumor region reveals marked peritumoral gliosis and proliferative tumor cells. (D) CD11b immunostaining shows extensive association of myeloid cells with tumor cells. Scale bars = 200 μm.

The ability of SapC-DOPS to target metastatic brain tumors was finally assessed in a second brain metastasis mouse model generated by intracarotid injection of NCI-H460 human large cell lung carcinoma cells. About 3 weeks after tumor cell injection (i.e., when signs of morbidity appeared) the mice were injected intravenously with SapC-DOPS-CVM and sacrificed 24 hs later. Similar to our metastatic breast cancer model, histological examination showed the presence of discrete micro- and macrometastases preferentially located in the hippocampus, thalamus, and midbrain (Fig. 9A). Examination of brain sections showed selective accumulation of SapC-DOPS-CVM in tumor cells (Fig. 9A). Compared with the brain metastases produced by breast cancer cells (Fig. 6E and 8B), the paucity of lectin-FITC signal within brain metastases of lung cancer cells indicated that these tumors were poorly perfused (Fig. 9B). Similar to the breast cancer metastasis model, lung cancer metastatic cells expressed phosphatidylserine (Fig. 9B), induced prominent gliosis (Fig. 9C) and were closely surrounded by CD11b-positive microglia/macrophages (Fig. 9D). These results indicate that SapC-DOPS CVM effectively and selectively target small metastases of human cancer cells in the mouse brain.

## DISCUSSION

Selective delivery of anticancer agents remains a key obstacle in implementing effective therapies for both primary and secondary, or metastatic, cancers. In the case of brain tumors, this problem is compounded by the presence of the blood brain barrier, which limits the passage of most drugs from the blood to the tumor stroma. We have shown that SapC-DOPS nanovesicles target tumor cells and exert antitumor actions both *in vitro* and *in vivo*, in mouse models of human neuroblastoma, malignant peripheral nerve sheath tumor [9], squamous cell carcinoma [19], GBM [11] and pancreatic cancer [18]. These studies suggested that tumor-selective binding and cytotoxicity effected by SapC-DOPS correlate with the ability of SapC to fuse with exposed membrane phosphatidylserine, which is abundant in many different cancer cells [14-16].

*Bioavailability and antitumor effects of SapC-DOPS in human glioblastoma mouse models.* We previously reported the antiangiogenic and pro-survival effects of SapC-DOPS on different mouse GBM models, including one that expressed EGFRvIII, a constitutively active EGFR representing the most common mutation found in EGFR-amplified GBMs [11]. In order to assess SapC-DOPS bioavailability after systemic administration, a critical factor for optimizing potential therapeutic interventions, in the present study we used the latter GBM mouse model to characterize the kinetics of SapC-DOPS accumulation. After a single intravenous injection, we observed a time-dependent accumulation of SapC-DOPS-CVM in intracranial U87ΔEGFR-Luc GBMs. In contrast, nanovesicles composed of DOPS-CVM failed to accumulate in the tumors, and tumor-free brain parenchyma showed no patent SapC-DOPS or DOPS uptake. As evidenced by vascular staining with fluorescent lectin, 24-48 hs after systemic injection SapC-DOPS-CVM accumulated in the extravascular space of U87ΔEGFR-Luc GBMs, indicative of its ability to cross the blood brain tumor barrier (BBTB). The high permeability of the tumor vasculature in this model, denoted by the patent extravasation of fluorescent, 70 kD dextran in the tumor mass, makes it possible that SapC-DOPS extravasation is aided by the “enhanced permeability and retention” effect [20]. However, because DOPS-CVM vesicles were not effectively taken up by tumor cells, the retention of SapC-DOPS within the tumor cannot be explained solely by the increased permeability of the BBTB. Rather, because systemically injected lactadherin (which binds phosphatidylserine with very high affinity, v.g. K_d_~0.08–4 nM) caused a marked blockade of SapC-DOPS-CVM targeting of GBM, we conclude that uptake and retention of SapC-DOPS by GBM cells *in vivo* is mediated by surface exposed phosphatidylserine. Taken together, these results confirm that SapC-DOPS crosses the BBTB and accumulate specifically in GBM in a PS-dependent fashion.

Considering GBM genetic heterogeneity, we next asked whether SapC-DOPS would also be effective against GBM tumors expressing the wild-type EGFR. Treatment of mice bearing subcutaneous human U87-MG xenografts with SapC-DOPS demonstrated significant tumor burden reduction, suggesting that SapC-DOPS may be therapeutically effective against GBM tumors with different genetic backgrounds.

*Targeting and cytotoxic effects of SapC-DOPS on metastatic brain tumor cells.* In the second part of our study, we explored the ability of SapC-DOPS to target, both *in vitro* and *in vivo,* breast cancer cells with brain metastatic potential. Its targeting efficacy toward human breast cancer MDA-MB-231-luc-D3H2LN cells was first evaluated in a co-culture system with normal, human astrocytes, a model that better resembles the microenvironment of brain tumors. After short incubation with fluorescently labeled SapC-DOPS, tumor cells bound and internalized significantly more SapC-DOPS than astrocytes. Assessment of PS levels by annexinV-FITC staining in these cell types showed that MDA-MB-231-luc-D3H2LN cells have ~3 times as much exposed phosphatidylserine than astrocytes; this finding provides a plausible mechanism for the observed selectivity of SapC-DOPS toward the tumor cells. The antitumor capacity of SapC-DOPS was confirmed through an *in vitro* cytotoxicity assay that showed that SapC-DOPS kills MDA-MB-231-luc-D3H2LN with an IC50 of 25.2 μM.

The capacity of SapC-DOPS to target brain metastases was then assessed in living mice. Two mouse models were generated by injecting MDA-MB-231-luc-D3H2LN cells, a strain of the MDA-MB-231 breast carcinoma cell line that has been shown to preferentially metastasize to the brain after intracardiac injection [21], or large cell lung carcinoma cells NCI-H460 [22], into the internal carotid artery of athymic mice [23, 24]. *In vivo* MDA-MB-231-luc-D3H2LN tumor luminescence in the brains was detectable 5-7 weeks after tumor cell injection. The progression of NCI-H460 tumors was faster, with animals showing signs of morbidity after 18-21 days.

Using histology and tissue immunofluorescence, we defined some morphological and molecular aspects of these metastatic brain cancer models. MDA-MB-231-luc-D3H2LN tumors were always multifocal and grew either alongside small blood vessels (vascular co-option) or as aggregates encasing dense capillary networks, suggestive of vascular remodeling. This strict association of tumor cells with vascular elements has been described in both animal models and human samples of brain metastases [22, 25, 26]. In contrast with MDA-MB-231-luc-D3H2LN tumors, NCI-H460 tumors were typically larger and fewer in number, had less vascular perfusion, and were not observed to grow in apposition to blood vessels.

Consistent with observations made in several human and experimental brain metastases [27-29], reactive glia, denoted by GFAP staining, surrounded micro- and macrometastatic clusters in both our models. Astrocytes have been shown to promote tumorigenesis by upregulating survival gene expression [30], reducing tumor-suppressor microRNA levels [31], stimulating cancer stem-cell proliferation [32], and facilitating the invasion [33] of metastatic cancer cells. We observed numerous nestin-expressing host cells in close proximity to metastatic MDA-MB-231-luc-D3H2LN cell clusters. Nestin is an intermediate filament expressed by neuroepithelial progenitor cells and newly formed endothelial cells and can be re-expressed by mature cells, such as astrocytes in glial neoplasms, and in response to brain injury [34-37]. We also show that brain metastases of MDA-MB-231-luc-D3H2LN cells express the VEGF receptor VEGFR1. Synthesized by most tumors and a primary mediator of tumor angiogenesis, VEGF has been shown to act autocrinally on MDA-MB-231 cells to promote growth [38, 39]. Moreover, the dependence on VEGF signaling for cancer progression has been demonstrated in a preclinical model of metastatic breast cancer to the brain [40]. The presence of CD11b-positive microglia/macrophages in close apposition to cancer cells in our two brain metastasis models reflects a common feature of brain malignant neoplasms [41]. Although both anti- and pro-tumoral actions for these tumor-associated macrophages have been suggested, there is a general agreement in that they play a permissive role in tumor growth. For instance, recent studies provided elegant evidence that microglia, the brain's resident immune cells, promote the invasion and colonization of brain tissue by breast cancer cells [33, 42].

After systemic injection, *in vivo* imaging showed that fluorescent SapC-DOPS-CVM nanovesicles colocalized with the luminescent signal of MDA-MB-231-luc-D3H2LN cells in the mouse brain, denoting selective targeting of metastatic tumor cells. Fluorescence microscopy of brain sections confirmed selective tumor targeting by SapC-DOPS in both breast and lung metastatic brain cancer models. Using immunofluorescence, we showed that phosphatidylserine expression was abundant in brain tumor cells in both mouse models; this fact, as discussed above, likely underlies the selective tumor targeting of SapC-DOPS. Interestingly, SapC-DOPS-CVM signal also overlapped with CD11b-positive myeloid cells in peritumoral regions characterized by hemorrhage and disrupted vascular integrity. The binding and uptake of SapC-DOPS by those cells should be favored both by the phagocytic activity of microglia/macrophages toward large macromolecules such as liposomes and by their expression of phosphatidylserine receptor(s). Previous studies have showed that SapC-DOPS effectively targeted inflammatory leukocytes in mouse models of arthritis [43] and affected the cytokine expression profile of macrophages [44]. In light of the important connection between inflammation and cancer, and recent reports indicating that antibody-mediated phosphatidylserine targeting promotes immune activation in diverse preclinical cancer models [45, 46], these observations suggest that SapC-DOPS, either alone or combined with a variety of drugs and ligands, may be useful to effect direct tumor cytotoxicity, to steer immune responses against tumor growth, and to modulate inflammatory reactions.

Finally, the antitumor effectiveness of SapC-DOPS was evaluated *in vivo* in our breast cancer metastasis model, showing that systemic treatment with SapC-DOPS resulted in extended survival. Our study presents some limitations. Because metastatic tumors in these mice do not arise from primary tumors, our models do not represent true metastases. However, they still recapitulate the essential steps of the metastatic process; i.e. hematogenous dissemination, vascular arrest and extravasation, and tumor cell growth. Importantly, many of the tumor-stromal relationships described in these models reproduce those encountered in the histopathological analysis of clinical specimens. Another limitation is that the study human tumor biology in preclinical models is so far restricted to, and conditioned by, the use of immunodeficient nude mice, and thus the anti-tumoral actions of the adaptive arm of the immune system are effectively excluded.

In conclusion, we have shown that SapC-DOPS nanovesicles can effectively target primary and metastatic brain tumors, and confer a significant survival advantage in a preclinical model of breast cancer metastasis to the brain. Systemically delivered SapC-DOPS nanovesicles are well tolerated and do not induce hepatotoxicity or manifest adverse effects [9]. We believe that the combined tumor selectivity and cytotoxic activity exhibited by SapC-DOPS equally *in vitro* and *in vivo* makes them attractive candidates for the diagnosis and treatment of brain tumors.

## MATERIALS AND METHODS

### Cell culture procedures and reagents

Human U87ΔEGFR-Luc glioblastoma cells, expressing a truncated, constitutively active, mutant epidermal growth factor receptor (EGFRvIII) were obtained from Dr. Webster Cavenee (Ludwig Cancer Institute, San Diego, CA). The U87-MG glioblastoma cell line was purchased from the American Type Culture Collection –ATCC (Manassas, VA, USA). Both cell lines were cultured in Dulbecco's modified Eagle's medium supplemented with 10% fetal bovine serum (FBS), 100U penicillin/ml, and 100 μg streptomycin/ml. Human breast carcinoma MDA-MB-231-luc-D3H2LN cells (Caliper Life Sciences, Mountain View, CA) were grown in Eagle's minimum essential medium with 10% FBS and penicillin/streptomycin (complete MEM). Human large-cell lung carcinoma cells NCI-H460 (ATCC HTB 177), purchased from ATCC (Manassas, VA), were cultured in RPMI-1640 medium supplemented with 10% FBS. Normal human astrocytes (ScienCell, Carlsbad, CA) were cultured in poly-L-lysine coated dishes in astrocyte medium (AM) that contained 2% FBS, growth factors, and antibiotics, according to instructions provided by the supplier. All cell lines were cultured at 37 °C in a 5% CO_2_ gas incubator.

### Fluorescent SapC-DOPS nanovesicle preparation

CellVue Maroon (CVM; Molecular Targeting Technologies Inc., Exton, PA) in ethanol was mixed with DOPS and SapC for bath sonication preparation as described previously [9]. Briefly, 82 μg DOPS (Avanti Polar Lipids, Alabaster, AL) were mixed with 30 μl CVM (from 1 mM stock) in a glass tube and the mix solvent evaporated under nitrogen gas. Human recombinant SapC protein [47] (0.4 mg) was added to DOPS-CVM along with 20 μl citrate/phosphate buffer pH 5.0. After addition of 1 ml PBS (or DMEM) and a 30 min sonication step, SapC-DOPS nanovesicles were separated from free CVM dye using a Sephadex G25 column (PD-10; Amersham Pharmacia Biotech, Piscataway, NJ). DOPS-CVM nanovesicles were prepared as described by omitting SapC.

### *In vitro* experiments

To establish co-cultures of astrocytes and MDA-MB-231-luc-D3H2LN tumor cells, astrocytes (passage number ≤ 4) were labeled with the PKH67 Green Fluorescent Cell Linker Kit (Sigma) and plated with tumor cells in media containing (1:1) AM:complete MEM.

For SapC-DOPS-CVM uptake experiments, cells were cultured in 12-mm diameter, poly-L-lysine-coated glass coverslips at a ratio of 1:1 astrocytes:tumor cells. After 48 hs, co-cultured cells were incubated with SapC-DOPS-CVM (125 ul; 3.5 uM SapC) in complete MEM for 30 min at 37^o^C. Cells were then washed with PBS, fixed in 4% paraformaldehyde and mounted in DAPI-containing aqueous mounting medium. CVM fluorescence was quantified from microphotographs (10 per individual culture) using Image J software (U. S. National Institutes of Health, Bethesda, Maryland, USA). Phosphatidylserine levels in cultured cells were determined by flow cytometry using an AnnexinV-FITC/PI staining kit (Life Technologies, Carlsbad, CA) according to the manufacturer instructions. SapC-DOPS cytotoxicity toward MDA-MB-231-luc-D3H2LN cells was evaluated using a MTT cell proliferation assay (Roche Diagnostics, Indianapolis, IN) in cells cultured for 72 h in the presence of various concentrations of SapC-DOPS (containing 4.5, 9, 22.5, or 45 μg SapC) or equimolar amounts of DOPS.

### Brain tumor mouse models and treatment protocol

All experiments involving mice complied with National Institutes of Health guidelines. Protocols were approved by the Institutional Animal Care and Use Committee of the University of Cincinnati (IACUC number 11-05-05-02) and the Cincinnati Children's Hospital Research Foundation (IACUC number 2013-0052). Orthotopic GBM xenografts were produced by stereotactic injection of 1 × 10^5^ U87ΔEGFR-Luc cells into 8 weeks old, anesthetized female athymic nude mice (Harlan), 2 mm lateral to bregma, at a depth of 3 mm. Sham-operated animals received a saline injection. Subcutaneous GBM xenografts were produced by injecting 1 × 10^6^ U87-MG cells into the dorsal right flank of 8-10 weeks old female athymic nude mice. Once the average tumor size reached ~100 mm^3^, mice (6 per group) received tail vein injections with a saline solution (Control) or SapC-DOPS (6mg/kg = SapC 4mg/kg, DOPS 2mg/kg) daily for the first 7 days, and then every 2 days for 10 days. Tumor size was assessed every 2-3 days by direct caliper measurement

The brain metastasis mouse models were created by injecting 1 × 10^5^ MDA-MB-231-luc-D3H2LN cells or 1 × 10^5^ NCI-H460 cells, in a total volume of 20 μl PBS, into the left internal carotid artery of female nude mice [23, 24]. To assess the therapeutic effect of SapC-DOPS, starting five days after intra-carotid injection with MDA-MB-231-luc-D3H2LN cells, mice were randomly distributed into 2 groups (8 mice per group) and administered SapC-DOPS (12mg/kg = SapC 8mg/kg, DOPS 4mg/kg) or DOPS (4mg/kg) nanovesicles. Injections (200 ul of SapC 0.8 mg/ml and/or DOPS 0.4 mg/ml in PBS) were given via tail vein daily for seven days, and then every two days for an additional ten days.

### In vivo bioluminescence and fluorescence imaging

For tumor imaging, mice were anesthetized with 2% isoflurane, and d-Luciferin (Caliper Life Sciences) at 150 mg/kg body weight was injected intraperitoneally 5 min before imaging. SapC-DOPS-CVM or CVM-labeled DOPS was administered by tail vein injection (200 μl volume) to mice bearing U87ΔEGFR-Luc or MDA-MB-231-luc-D3H2LN brain tumors. Images were taken using an IVIS 200 Series (Clipper, Alameda, CA) or a Kodak FX (Carestream Health, Toronto, Ontario, Canada) imaging system.

### Vascular staining and permeability, lactadherin administration, and CVM quantification

Staining of mouse brain vasculature was done by injecting 100 μg of tomato lectin-FITC (Vector Laboratories, Burlingame, CA) into the tail vein 2 min before sacrifice. To assess vascular permeability, mice were similarly injected with 1 mg TRITC-dextran 70,000 MW (Life Technologies, Carlsbad, CA) 30 min before sacrifice. Bovine lactadherin (Haematologic Technologies, Inc, Essex Junction, VT) was injected intravenously (10 μg) in saline. Dissected brains were frozen in dry ice and stored at -80 ºC. After embedding in OCT freezing medium, cryosections (25 μm) were mounted in glass slides using DAPI-containing medium. Images were obtained in a confocal fluorescence microscope (LSM 710; Carl Zeiss) using appropriate filters. Brain CVM fluorescence was registered by exciting at 633 nm. Quantification of CVM-labeled SapC-DOPS or DOPS signal in mouse GBM sections was performed using ZenLite software (Carl Zeiss).

### Immunofluorescence

Frozen brain sections (8-10 μm) were fixed in 2% paraformaldehyde, blocked with Power Block (Biogenex), and incubated with rabbit anti- glial fibrillary acidic protein (GFAP; 1:100; Cell Signaling Technology, Danvers, MA), mouse anti-nestin (1:10; Rat-401; Developmental Studies Hybridoma Bank, University of Iowa) or rabbit anti-Ki-67 (ScyTek) for 24 hs at 4ºC. Alternatively, frozen sections were air-dried, blocked with 5% normal goat serum, and incubated with mouse anti-phosphatidylserine antibody (1:100; clone 4B6; Abcam); rat anti-CD11b (1:10; M1/70.15.11.5.2; Developmental Studies Hybridoma Bank); rabbit anti-vascular endothelial growth factor receptor 1 (VEGFR1; 1:100; Bioss, Woburn, MA), or mouse anti-bovine lactadherin (1:5; L688; Developmental Studies Hybridoma Bank) for 24 hs at 4ºC. Species-specific, Alexa-488 or Alexa-555-conjugated, goat secondary antibodies (Cell Signaling Technology) were used to label primary antibodies. A biotinylated anti-rabbit antibody followed by Streptavidin-FITC staining was used to detect VEGFR1. Incubations were carried on in PBS containing 25% glycerol. Microphotographs were taken with either a LSM 710 confocal microscope or an Olympus BX51epifluorescence microscope.

### Statistical analysis

GraphPad Prism software was used to compare Kaplan–Meier curves using the log-rank test. SapC-DOPS-CVM uptake in cultured cells and *in vivo* tumor growth were analyzed using two-tailed, unpaired Student's t tests. A P value <0.05 was considered statistically significant. All error bars represent standard error (SE).
